# Understanding Social Contagion in Adoption Processes Using Dynamic Social Networks

**DOI:** 10.1371/journal.pone.0140891

**Published:** 2015-10-27

**Authors:** Mauricio Herrera, Guillermo Armelini, Erica Salvaj

**Affiliations:** 1 Facultad de Ingeniería, Universidad del Desarrollo, Santiago, Chile; 2 ESE Business School, Universidad de los Andes, Santiago, Chile; 3 Facultad de Economía y Negocios, Universidad del Desarrollo, Santiago, Chile; IFIMAR, UNMdP-CONICET, ARGENTINA

## Abstract

There are many studies in the marketing and diffusion literature of the conditions in which social contagion affects adoption processes. Yet most of these studies assume that social interactions do not change over time, even though actors in social networks exhibit different likelihoods of being influenced across the diffusion period. Rooted in physics and epidemiology theories, this study proposes a Susceptible Infectious Susceptible (SIS) model to assess the role of social contagion in adoption processes, which takes changes in social dynamics over time into account. To study the adoption over a span of ten years, the authors used detailed data sets from a community of consumers and determined the importance of social contagion, as well as how the interplay of social and non-social influences from outside the community drives adoption processes. Although social contagion matters for diffusion, it is less relevant in shaping adoption when the study also includes social dynamics among members of the community. This finding is relevant for managers and entrepreneurs who trust in word-of-mouth marketing campaigns whose effect may be overestimated if marketers fail to acknowledge variations in social interactions.

## Introduction

Social network theory holds great importance for understanding social influences. Ideas, information, trends, opinions, adoptions of new technologies, fads, and many other human activities spread among individuals through social interactions [1, 2], which makes seeking to understand how social influence works and the extent to which it affects adoption, highly relevant.

Early research assumed temporal and spatial homogeneity in social influence (i.e., every actor had the same probability of influencing or being influenced [3]). However, people interact with varying numbers of others, and with some of them more frequently than with others. Research that accounts for such heterogeneity in human relations started using network models [4, 5] to address issues such as the role of opinion leaders in diffusion processes [5], in finding out how to encourage people to get the word out [6], to determine whether regular consumers encourage the diffusion of innovations [7], and to discover whether there is a complementary or substitute effect between social influence and traditional marketing (e.g., advertising) [5, 8].

Modeling social contagion using network models requires taking two conditions into account [9]. First, researchers must identify and follow up on users of the new product or innovation over time, because individuals might adopt or reject the innovation at different times. Second, they must account for variations in social relations among the community of customers over time, since social interactions are inherently unstable and change frequently. In other words, the contact pattern among individuals does not have a static architecture, but rather coevolves with the population as the contagion progresses.

Research in social interaction has distinguished between two types of dynamic networks. Those networks in which the topology changes regardless of the contagion process taking place on them are called evolving networks, while those networks that change their topology to mitigate or promote these processes are called adaptive networks [10]. During recent years adaptive behavior in contagion has been studied extensively [11–23]. An adaptive case happens when the behavioral changes produced by the perception of risk during the contagion process modify the network structure. Given the wide range of human reactions and its impact on the spread of the contagion, several models have been developed (See a review in [15]).

In adaptive networks, the social dynamic has been modeled using different types of rewiring methods. For instance, as reported in [16, 21] once an uninfected vertex loses connection with an infected neighbor it establishes a new connection only with other susceptible vertices. This sort of rewiring is appropriate for symptomatic contagion. On the other hand, [18] reports a new connection might be established at random with any other vertex without discrimination. In [14] preferential reconnections take place only among infected vertices reflecting a sort of self-imposed quarantine. An adaptation phenomenon is considered in [10] when an uninfected vertex, based on local information, is disconnected from an infected vertex with a certain probability and restores it after a fixed time. In conclusion, adaptive network models have contributed to our understanding of dynamic networks because they provide insight into the qualitative and universal features of how adaptive behavior affects the dynamics of contagion.

Our empirical setting and data correspond to an evolving network. In this regard we follow mainly the work of Schwarzkopf et. al. [11] which accounts for an indiscriminate rewiring, independent of the contagion process. Additionally, the studies previously mentioned are based mostly on some theoretical assumptions and extensive computer simulations, while our approach, is more closely related to data-driven model methodologies and is intended to describe real processes based on rich and detailed longitudinal datasets.

Concerning the studies reported in the marketing and diffusion of innovation literature, extant models, probably due to a lack of information, account for only the variation in adopters over time, using a single social network across time (e.g. [4, 5]). As a result, the prevalence of social contagion in the adoption process might be over or underestimated, because of not considering network dynamics. To the best of our knowledge, this issue remains unaddressed, despite its important implications for marketing and diffusion of innovation theory. For example, the assumption that social contagion is at work has prompted many entrepreneurs and small firm owners with scanty resources to rely on word-of-mouth or grass-roots marketing strategies to promote their products or services. But it could be that managers are overestimating the power of social contagion; these word-of-mouth (WOM) strategies might not be working as well as expected.

In summary, the central research question addressed in this paper is to understand how social dynamic in evolving networks affect the weight of social contagion in the process of adoption of new products.

## 1 Materials and Methods

### 1.1 SIS Model in a Static Network

To model social contagion, we use a SIS approach, which is common in physics, epidemiology, and diffusion of innovation research [7, 23–26]. The models can depict contagion of individual entities, who switch from potential customers to adopters and vice versa. They have also been used to analyze the spread of diseases in heterogeneous networks with arbitrary vertex distribution (e.g., [27]).

In our model, those who have adopted an innovation are “infected”. The fraction or density of the population in an infected state at time *t* is represented by *x*(*t*). Non-adopters (individuals *at risk* of adopting) are in the susceptible state *s*(*t*) at time *t*. Each person in a susceptible state *s*(*t*) at a time *t* is coded as either having transitioned to the infected state *x*(*t*) (*s*(*t*) → *x*(*t*)) or not prior to this time (similar to transitions of the type *x*(*t*) → *s*(*t*)).

As an efficient means of constructing an SIS model, we applied mean field (MF) theory. Such models can reveal the behavior and interactions of a large number of individual components, thereby reducing complex problems to simpler ones and, in some cases, providing very accurate results for real-world networks.

Two conditions are necessary to enable MF models to work better in real-world networks:

1. The states of the neighbor vertices must be independent of each other. Clustering coefficients and transitivity in the network should be low. For low transitivity networks, the vertex states (susceptible or infected) and those of their neighbors can be treated as independent, in such a way that it is possible to neglect *dynamic correlations*.
2. The average connectivity of the vertex neighbors should exceed the average connectivity of the vertices. MF theory produces relatively few errors for networks in which the vertices have highly connected neighbors [28].

An interesting consequence for random mixing networks (see Section 3.3) is that the average connectivity of the individuals’ neighbors exceeds the average connectivity of the individuals. Therefore, the MF model should give very accurate results for these types of networks.

The *degree* of a vertex is defined as the number of edges that enter or exit from it. We propose using the degree as the network unit of analysis. Such a degree-based approximation was first proposed by Pastor-Satorras [27, 29–31], under the basic assumption that all vertices in a given degree class can be considered to be statistically equivalent. That is, all vertices of the same degree have the same probability of contagion at any given time. This approach takes its simplest form when applied to a network with a given distribution function. In this case, the results are equivalent as to work at the vertex level but are computationally more efficient.

Let *s*_*k*_(*t*) and *x*_*k*_(*t*) be the densities of susceptible and infected vertices with exact *k* connections (degree), respectively. The degree distribution of a network can be described by *P*_*k*_, which is the fraction of vertices with degree *k*. Then the densities *s*_*k*_(*t*) and *x*_*k*_(*t*) can be defined as follows:

$$\begin{array}{ccc} s(t) & = & \sum_{k}P_{k}s_{k}(t)\\ x(t) & = & \sum_{k}P_{k}x_{k}(t) \end{array}$$

Furthermore, these variables obey the following normalization condition:

$$\begin{array}{c} s_{k}+x_{k}=1 \end{array}$$

To assess the transmission of contagion, we use the *excess degree* of a vertex [24, 32]. If we follow an edge to a vertex, the excess degree is one less than its degree. Contagion cannot be transmitted back along the edge from which it came. The probability of reaching a vertex of degree *k* (or the excess degree *k* − 1) by following a random edge is therefore proportional to *k*. In turn, the probability that a vertex at the end of a random edge has an excess degree *k* − 1 is a constant multiple of *kP*_*k*_, with the constant chosen to ensure that the sum over *k* of the probabilities is equal to 1. Then the probability that a vertex has excess degree *k* − 1 is $q_{k}=\frac{kP_{k}}{\langle k\rangle }$, where the mean degree 〈*k*〉 serves to ensure the normalization of the distribution. This distribution also characterizes degree-uncorrelated networks.

The adoption process might occur for two reasons:

1. Non-social factors. To include these factors in the model, we use the term *ϑ* *s*_*k*_(*t*), which indicates the spontaneous contagion of the susceptible population at a constant rate *ϑ* [33]. This parameter captures all non-social issues that might drive adoption, such as firm advertising, the diffusion of the product in other countries, pricing, and so forth.
2. Contact with an infected infectious vertex through the links of the social network. This probability is [27, 29, 31]:
  $$\begin{array}{c} \Theta_{k}(t)=\sum_{h}P\left(h|k\right)x_{h}\left(t\right) \end{array}$$
  More precisely, Θ_*k*_(*t*) is the probability that an edge emanating from a vertex of degree *k* points to an infected vertex. Here, *P*(*h* ∣ *k*) is the conditional probability that a vertex of degree *k* is connected to a vertex of degree *h*. However, in the case of an uncorrelated random network, the probability that an edge points to a vertex with *h* connections coincides with the probability that this vertex has excess degree *h* − 1, such that
  $$\begin{array}{c} P\left(h|k\right)=\frac{hP_{h}}{\langle h\rangle } \end{array}$$
  where we rely on the knowledge that 〈*k*〉 = 〈*h*〉. Then,
  $$\begin{array}{c} \Theta (t)=\sum_{h}P\left(h|k\right)x_{h}\left(t\right)=\frac{1}{\langle h\rangle }\sum_{h}hP_{h}x_{h}\left(t\right) \end{array}$$ (1)
  In this case, Θ(*t*) is independent of the degree *k*. Next, we define *β* as the transmission rate at which a susceptible individual changes from a susceptible to an infected state after contact with a single infected vertex. With a probability *β* per time unit, infected vertices spread the contagion to their susceptible neighbors.
  The probability of transmission from any neighbor of one particular vertex is *β*Θ(*t*)*dt*. In addition, this particular vertex must itself be susceptible, which happens with probability *s*_*k*_(*t*), so the final probability that the vertex will become infected is *s*_*k*_(*t*)*β* *k*Θ(*t*)*dt*. The factor *k* stems because each network vertex admits the transmission from each of its contacts. Thus, the rate of change of *s*_*k*_ ($\frac{d}{dt}s_{k}(t)$) due to social contagion is given by −*β* *k* *s*_*k*_(*t*)Θ(*t*)

In addition, we consider the parameter *γ* representative of the recovery rate of an individual from the infected state *x*_*k*_ and thus its return to the susceptible state *s*_*k*_. To include this recovery, we use the term *γ* *x*_*k*_(*t*).

Taking all these considerations into account, we derive the following model, which we will denote by SIS_*ϑ*_ model:

$$\begin{array}{ccc} \frac{d}{dt}s_{k}(t) & = & -\beta \;k\;s_{k}(t)\Theta (t)-\vartheta \;s_{k}(t)+\gamma \;x_{k}(t)\\ \frac{d}{dt}x_{k}(t) & = & -\gamma \;x_{k}(t)+\beta \;k\;s_{k}(t)\Theta (t)+\vartheta \;s_{k}(t) \end{array}$$

Table 1 describes the parameters and variables of the model.

**Table 1:** Parameters and Variables of the Model.

Model Parameters	
*β*	Transmission rate due to social influence.
*ϑ*	Spontaneous adoption rate due to external influences
*γ*	Recovery rate
Model Variables	
*s*_*k*_(*t*)	Density of susceptible individuals (non-adopters) with degree *k*
*x*_*k*_(*t*)	Density of infected individuals (adopters) with degree *k*
Θ(*t*)	Probability that an edge from a vertex of degree *k* points to an infected vertex

By replacing *s*_*k*_ = 1 − *x*_*k*_ in the equation for *x*_*k*_, we obtain:

$$\begin{array}{c} \frac{d}{dt}x_{k}(t)=-\alpha \;x_{k}(t)-\beta \;k\;x_{k}(t)\Theta (t)+\beta \;k\;\Theta (t)+\vartheta \end{array}$$ (2)

where *α* = *ϑ*+*γ*.

### 1.2 Modeling the Dynamics of the Network

In model (2), we assumed that network relations did not vary over time. However, when the number or identity of neighbor vertices in a particular vertex changes, each vertex is no longer connected to a static set of neighbors but to a dynamic one. To assess this social network dynamic, we measure the topological overlap between graphs in the time series. We use a variation on the temporal correlation coefficient presented in [34], in such a way that our variation corrects for the large number of temporal disconnected vertices in the time series. Therefore, as defined in [34, 35] the *topological overlap* in the neighborhood of *i* between two consecutive time steps [*t*_*m*_, *t*_*m*+1_] is:

$$\begin{array}{c} C_{i}\left(t_{m},t_{m+1}\right)=\frac{\sum_{j}a_{ij}\left(t_{m}\right)a_{ij}\left(t_{m+1}\right)}{\sqrt{\left[\sum_{j}a_{ij}\left(t_{m}\right)\right]\left[\sum_{j}a_{ij}\left(t_{m+1}\right)\right]}} \end{array}$$ (3)

where *a*_*ij*_ represents an entry in the unweighted adjacency matrix of the graph. Therefore, summing over *a*_*ij*_ gives the edges between *i* and every other vertex. Next, the *average topological overlap* of the graph at *t*_*m*_ with the subsequent graph at *t*_*m*+1_ is given by:

$$\begin{array}{c} C_{m}=\frac{1}{\max[N\left(t_{m}\right),N\left(t_{m+1}\right)]}\sum_{i=1}^{N}C_{i}\left(t_{m},t_{m+1}\right) \end{array}$$ (4)

Here, we average the maximum number of vertex participants in the network, instead of the total number. The metric suggested in [34, 35] calculates averages by a fixed *N*, or the total number of vertices in the network, so the resulting value underestimates the topological overlap by the percentage of non-participating vertices. Further discussion of the difference between this metric and the one presented in [34, 35] is available in [36]. The average temporal overlap between any two temporally adjacent graphs (*temporal correlation coefficient*) in a series is given by:

$$\begin{array}{c} C=\frac{1}{M-1}\sum_{m=1}^{M-1}(\frac{1}{\max[N\left(t_{m}\right),N\left(t_{m+1}\right)]}\sum_{i=1}^{N}C_{i}\left(t_{m},t_{m+1}\right)) \end{array}$$ (5)

If coefficient *C* equals 1, the network is static. If *C* is less than 1, we should use network models that account for network variability. We apply Eq (5) to our data in section 3.5 to verify the constant turnover of neighbor vertices in the network.

Fefferman and Ng in [37] show that continuous shifting of social contacts based on individual association preferences powerfully affects social contagion processes. On the one hand, the local network dynamic can define consistent properties of the convergent stationary network structure (e.g., well-defined degree distribution function) [38, 39]. On the other hand, it will affect the contagion processes even after the network has reached a stationary or stable state. Thus, to investigate the potential role of individually driven network dynamics in social contagion, we should consider that the social network is not static but rather changes over time.

To capture social interaction among individuals, we opt for a rewiring model. Given that our empirical setting is characterized by an evolving network, our approach accounts for indiscriminate rewiring, independent of the contagion process, but dependent on the vertex degree.

A general indiscriminate rewiring dynamic is as follows: an edge is chosen at random and one end of the edge initially connected to vertex *k* is rewired to another vertex *k*′ with rate *u*(*k*, *k*′). This is a random and instantaneous process. During the rewiring the number of vertices *N* and the number of edges *E* are unchanged but the edges are stochastically detached from one vertex and reattached to another.

A first simplification, is for *u*(*k*, *k*′) to factorize into a function of *k* and a function of *k*′, thus the overall rate of a rewiring event such that two vertices with degrees *k* and *k*′ end up with degrees *k* − 1, *k*′+1 is *u*(*k*, *k*′) = *kϕ*_*k*_ *v*_*k*′_. The factor *k* stems from choosing an edge at random. Writing *u*_*k*_ = *kϕ*_*k*_ we have the rewiring rate *u*(*k*, *k*′) = *u*_*k*_ *v*_*k*′_ where *u*_*k*_ is the detachment rate and *v*_*k*′_ the attachment rate.

**Fig 1:**
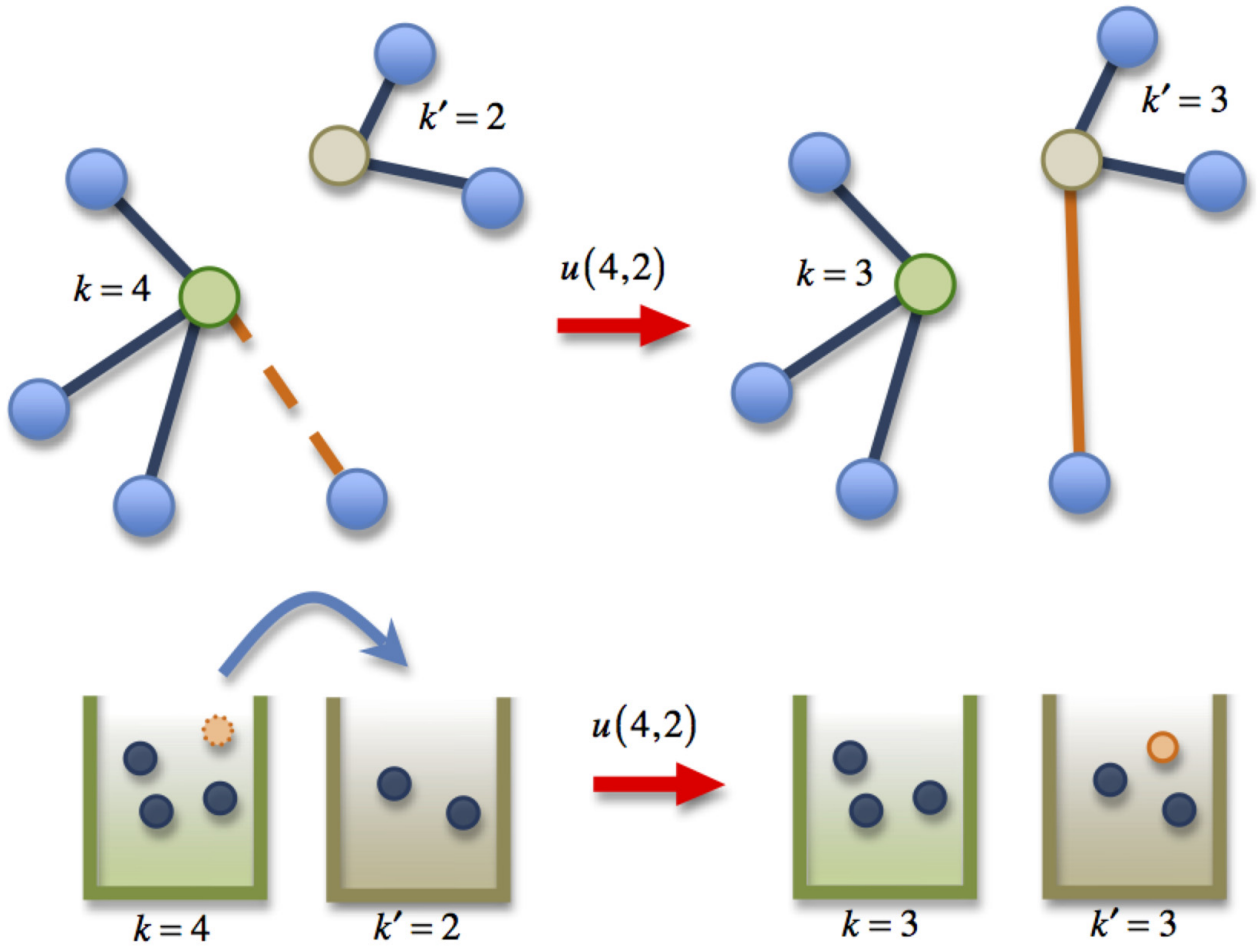
The Mapping of the Misanthrope Process to Rewiring Dynamics in Networks.

In order to better understand the indiscriminate rewiring process we map network rewiring dynamics to the dynamics of hopping particles (see Fig 1). Thus, we think of the number of edges connected to a vertex (vertex degree) as the number of particles at a site. The rate at which a particle hops from a site with *k* to a site with *k*′ particles is then *u*(*k*, *k*′) = *u*_*k*_ *v*_*k*′_. Using the particle interpretation, *u*_*k*_ and *v*_*k*_ are respectively the rates at which a particle leaves a generic site (*k* → *k* − 1), or arrives in this site (*k* → *k* + 1). These models are known as *driven diffusive systems* [40]. A particular driven diffusive system is the *Misanthrope process* [41] in which the hopping rate *u*(*k*, *k*′) depends on the occupancy of both the departure and target sites. Since an edge may be rewired from one vertex to any other in the indiscriminate rewiring process, we are considering Misanthrope dynamics on a fully connected geometry [42]. In this regard, given that we use a mean—field approach, fully connected geometry or mean—field geometry is guaranteed [43].

The network configuration at time *t* is determined by the degree sequence *K*_*i*_(*t*) (the occupation numbers in the particle-simile) with *i* = 1…*N*, and *N* the number of network vertices [32]. We assume that the probability of finding the network in a given configuration at time *t* factorizes, i. e. *P*(*K*_1_, *K*_2_, …, *K*_*N*_) = *P*(*K*_1_)*P*(*K*_2_)…*P*(*K*_*N*_). Factorization is a crucial issue because it allows focusing on the probability that an arbitrary vertex *i* has degree *k*, *P*_*k*_(*t*) = *P*(*K*_*i*_(*t*) = *k*) (in the particle-simile, the probability of finding *k* particles in the generic site *i*).

The probability *P*_*k*_(*t*) (that a site contains *k* particles at time *t*) is the degree distribution and it is also the fraction of vertices in the network with degree *k*. *P*_*k*_(*t*) satisfies the master equations given by [38]:

$$\frac{d}{dt}P_{k}\left(t\right)=u_{k+1}P_{k+1}+v_{k-1}P_{k-1}-\left(u_{k}+v_{k}\right)P_{k},\;k\geq 1$$ (6)

$$\frac{d}{dt}P_{0}\left(t\right)=u_{1}P_{1}-v_{0}P_{0}$$ (7)

These equations are non-linear, due to the dependence of the rates *u*_*k*_ and *v*_*k*_ of *P*_*k*_(*t*). The equation for *P*_0_ is special because one cannot select a zero degree vertex (an empty site) as a departure vertex (site).

### 1.3 The SIS_*ϑ*_ Model with Rewiring

Following Schwarzkopf et al. [11] we consider a generalization of the model (2) with rewiring (a detailed derivation of this model is included in the appendix) in the form:

$$\begin{array}{c} \frac{d}{dt}x_{k}=-\alpha \;x_{k}-\beta \;kx_{k}\Theta +\beta \;k\Theta +\vartheta -\;x_{k}(u_{k}+v_{k})+(\frac{x_{k+1}P_{k+1}u_{k+1}}{P_{k}}+\frac{x_{k-1}P_{k-1}v_{k-1}}{P_{k}}) \end{array}$$ (8)

The Eq (8) takes the change in the densities of infected vertices with degree *k* due to the rewiring dynamics into account. Thus, when an edge end of an infected vertex (with a rate of *v*_*k*_) attaches to a vertex of degree *k*, or else when a vertex of degree *k* is detached (with a rate of *u*_*k*_), the number of infected vertices of degree *k* decreases. This loss is described by the term − *x*_*k*_(*u*_*k*_ + *v*_*k*_) in model (8). On the other hand, the gain term $\;(\frac{x_{k+1}P_{k+1}u_{k+1}}{P_{k}}+\frac{x_{k-1}P_{k-1}v_{k-1}}{P_{k}})$ in model (8) reflects infected vertices of degree *k* − 1 that get an edge and the infected vertices of degree *k* + 1 that lose an edge. Both cases ultimately arrive at a state with *k* edges.

It is worth noting that three points differentiate our study from [11]. First, we include an extra term that accounts for spontaneous contagion. Second, the empirical network under analysis is not a free scale network. And finally, attachment and detachment rates are estimated directly from the data, fitting theoretical curves from the model (8) with the one of the evolution of adoptions over time.

## 2 Data Analysis

### 2.1 Social Network Data

The data set consists of records of telephone calls between community members in a small town (approximately 4000 inhabitants). We have information on telephone calls among the entire database of customers (corresponding to 1147 households) of a particular phone service between 1998 and 2007, on the base of one month. To build the social network, we used the phone numbers as labels for network vertices and calls as proxies for contacts or edges between these vertices. Phone networks previously have been used to depict the communication and social network contact among members of a community (e.g., in [44, 45]). The raw data are lists of the total phone calls between vertices in a given month for a span of ten years. These lists are directed (i.e., *i* calls *j* differs from *j* calls *i*) and aggregated monthly (the sum of all calls from *i* to *j* in one month). The graphs and corresponding adjacency matrices created from these lists are weighted and directed.

We discard all links that are not bidirectional from the original graph and treat the remainder as undirected edges. Yet not all directed links correspond to a real social tie: For example, *i* might consider *j* her friend, but *j* may not have that same consideration of *i*. Also, the directionality of the link between two vertices in the graph does not indicate any sort of hierarchical or differential social status among the community members in this case. Before performing any analysis, we must therefore make the graphs undirected. If the relationship between two vertices is not mutual in a given month, the edge is removed from the graph. Thus, two vertices are connected with an undirected edge if there were at least one pair of reciprocated phone calls between them (i.e., *i* calls *j*, and *j* calls *i*). Approximately 57% of the edges are excluded from the graph with this step.

### 2.2 Internet Adoption Data Over Time

We studied the diffusion process of Internet service. We have data on the time of adoption of this service from 1998 to 2007 for 250 customers (right censored). The raw *adoption* data for Internet services is given as a *Y* matrix with dimension *N* × *T*, where *N* = 1147 is the number of vertices, and *T* = 118 is the number of months in the observation period. If a vertex has Internet service in a given month, the corresponding matrix entry is equal to 1; a 0 would denote no Internet service.

**Fig 2:**
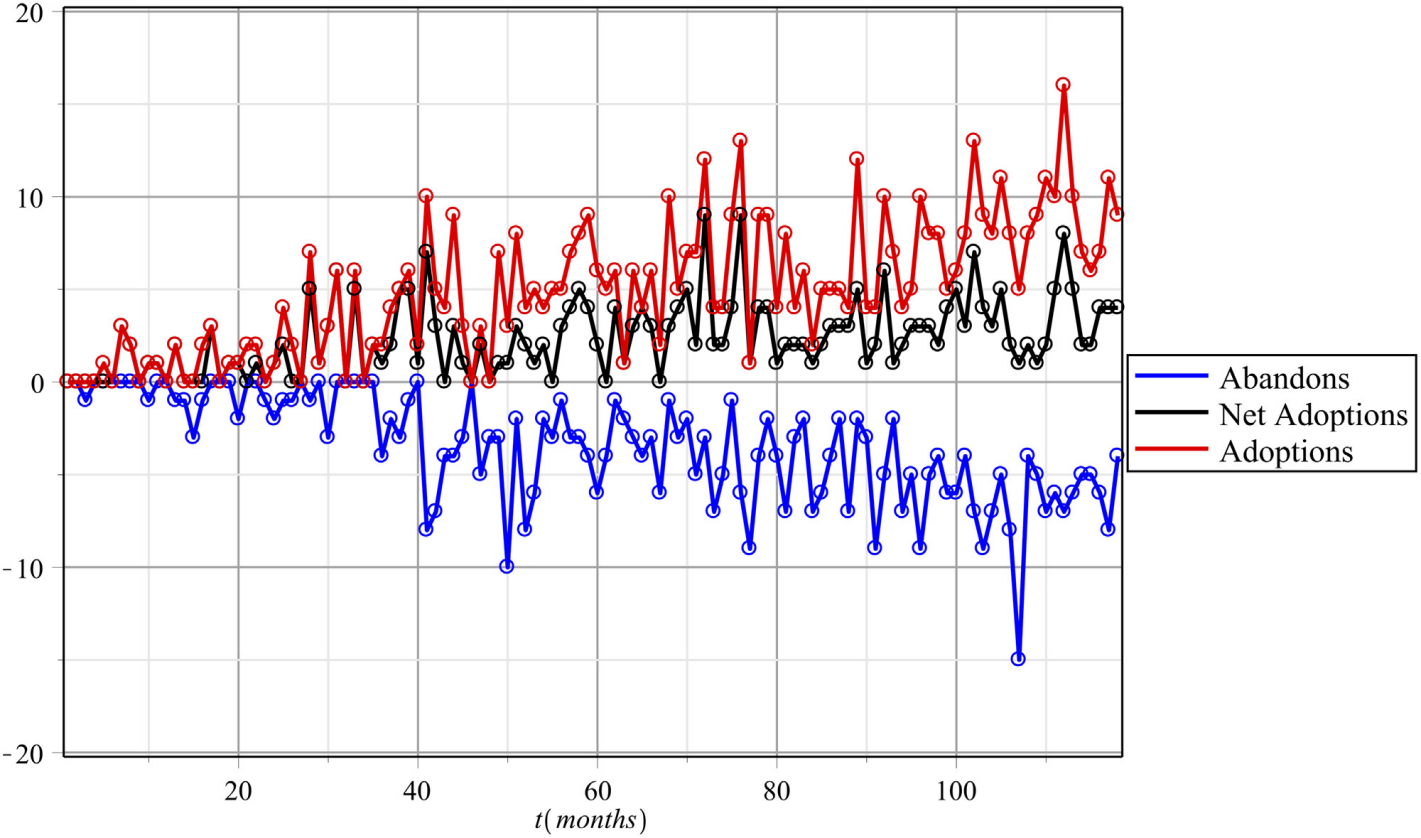
Fluctuations per month. The red line indicates the number of people who transitioned from susceptible to infected (0 → 1); the blue line reveals the number who transitioned from infected to susceptible (1 → 0); and black shows the net change in the total number of adoptions for the given month.

The adoption state may be acquired and lost repeatedly. An individual who ceases to be an adopter immediately becomes susceptible (i.e., a consumer *at risk* of adopting). The fluctuations between 1 → 0 → 1 are common in the time series, and we account for them by treating 0 users as *susceptible* entities (see Fig 2). A first-time adoption is the first time a vertex adopts, and each vertex can appear once in a calculation involving first-time adoptions. Generally, adoption implies a 0 → 1 transition, and a vertex can transition more than once.

In our empirical investigation we are dealing with an evolving network. People do not change connections with other individuals because they have contracted Internet service. In other words, Internet spread does not alter the structure of contacts. However, the social interaction within the community changes over time and consequently affects the spread of the Internet in the population.

## 3 Network Structure Over Time

To study adoption processes across evolving social networks, and in line with our model assumptions, we must characterize the structure of the network in which adoption may take place.

### 3.1 Degree Distribution

**Fig 3:**
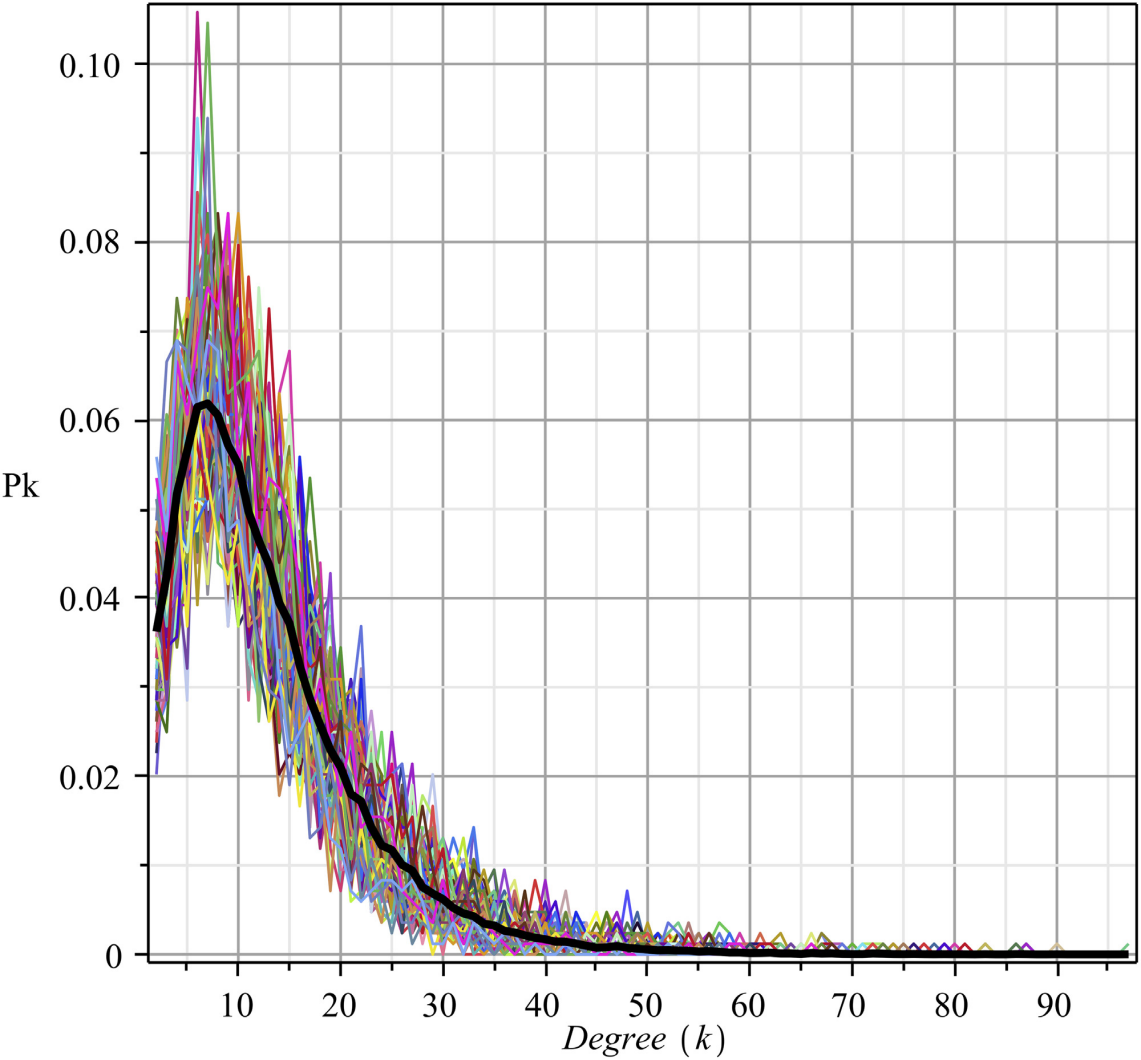
Degree probability function of the social network over time. The average probability distribution function (PDF) is in black. This PDF came from fitting by *Pk* ~ *k^a^e^−bk^*. The coefficients (95% confidence bounds) are as follows: *a* = 0.8199(0.7873, 0.8525), *b* = 0.1548(0.1506, 0.1589); goodness of fit (SSE) = 0.777 × 10^−4^; R-square = .9975, adjusted R-square = .9975, RMSE = 0.9141 × 10^−3^.

Fig 3 depicts the degree distribution functions, calculated over several months.

Although the observation window for registering the data is very large (ten years), the degree distribution function does not reveal many changes. On average, it retains the same shape over time (see Fig 3). This empirical evidence indicates that while the social network is in a steady state, the monthly distribution function fluctuates around an average distribution. This very desirable feature of the network allows for a comparison of the vertex degree across months. Moreover, for future calculations, the degree distribution function *P*_*k*_(*t*) of a particular month *t* may be replaced by the average degree distribution function *P*_*k*_, taken over all the months in the observation window.

In addition, the mean degree 〈*k*〉, defined as 〈*k*〉 = ∑_*k*_ *kP*_*k*_, of the participating vertices (vertices with at least one link) in the graph remains relatively constant (a line of best fit has a slope of 0.01). Variation between months is much less than variation between vertices: the sample standard deviation of the mean degree of participating vertices between months is 0.85, whereas the standard deviation of the mean degree over all the months between vertices is 7.32. However individual vertices show significant variation in degree over time.

### 3.2 Condition 1 of the MF Model: Clustering Coefficient

To check condition 1 from Section (1.1), we measured the clustering (transitivity) coefficient, which can indicate what fraction of the vertices in a graph are involved in triangles [46]. It is calculated as:

$$\begin{array}{c} c_{i}=\frac{1}{k_{i}\left(k_{i}-1\right)}\sum_{j,h}a_{ij}a_{ih}a_{jh} \end{array}$$ (9)

where *k*_*i*_ is the degree of vertex *i*.

**Fig 4:**
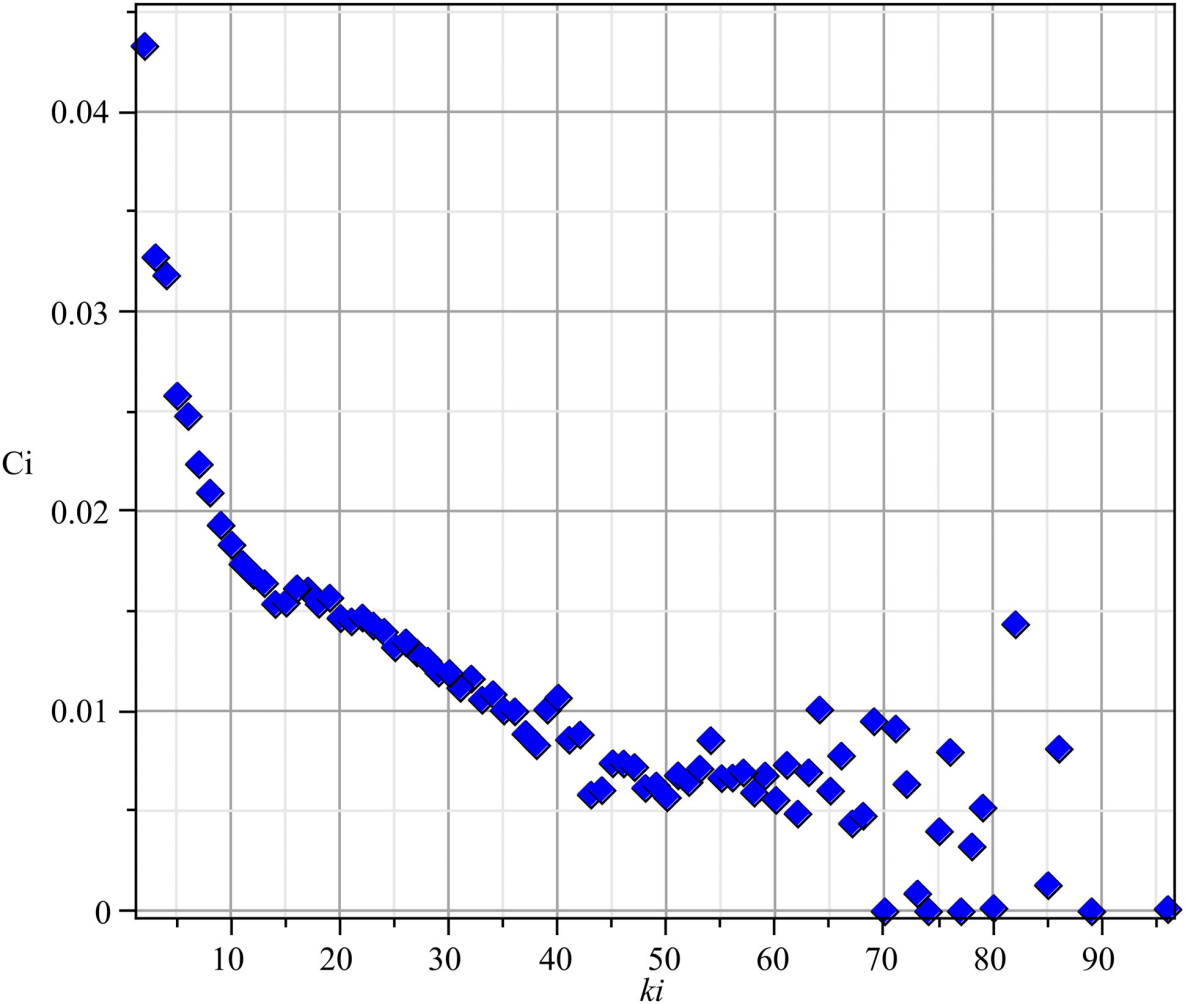
Average clustering coefficients (*c*_*i*_) versus vertex degree (*k*_*i*_), for each vertex and calculated for the time series of networks.

Fig 4 presents the average clustering coefficient, depending on the vertex degree. As shown, the clustering coefficient for the vertex in the network is very low and falls even lower as the vertex degrees increase.

### 3.3 Condition 2 of the MF Model: Average Nearest Neighbor Degree *k*_*nn*_ and Degree Assortativity

To confirm condition 2 from Section (1.1), we studied the *average nearest neighbor degree* of vertices of degree *k*, as defined by $k_{\text{nn}}(k)=\sum_{h}hP(h|k)$ over time. In this case, *P*(*h* ∣ *k*) is the conditional probability that a vertex of degree *k* is connected to a vertex of degree *h*.

If two-point correlations are not present in the network, the conditional probability takes the form $P\left(h|k\right)=\frac{hP_{h}}{\langle k\rangle }$, and the average nearest neighbor degree reads:

$$\begin{array}{c} k_{\text{nn}}=\sum_{h}\frac{h^{2}P_{h}}{\langle h\rangle }=\frac{\langle h^{2}\rangle }{\langle h\rangle }=\langle h\rangle +\frac{Var(h)}{\langle h\rangle } \end{array}$$ (10)

which is independent of *k*.

In this case, we encounter *proportionate mixing*, also known as *random mixing*, which occurs when vertex interactions have no particular preference. Connections are seemingly made at random from the connection pool, with a probability distribution function $q_{h}=\frac{hP_{h}}{\langle h\rangle }$ [47]. As a consequence of proportionate mixing, the average connectivity of the individuals, neighbors exceeds the average connectivity of the individuals, as Eq (10) indicates.

In contrast, the explicit dependence of $k_{\text{nn}}(k)$ on *k* necessarily implies the existence of non-trivial correlations. For example, in many social networks, vertices with high degrees more preferably connect to highly connected vertices, reflecting a property referred to as *assortative mixing* [46].

**Fig 5:**
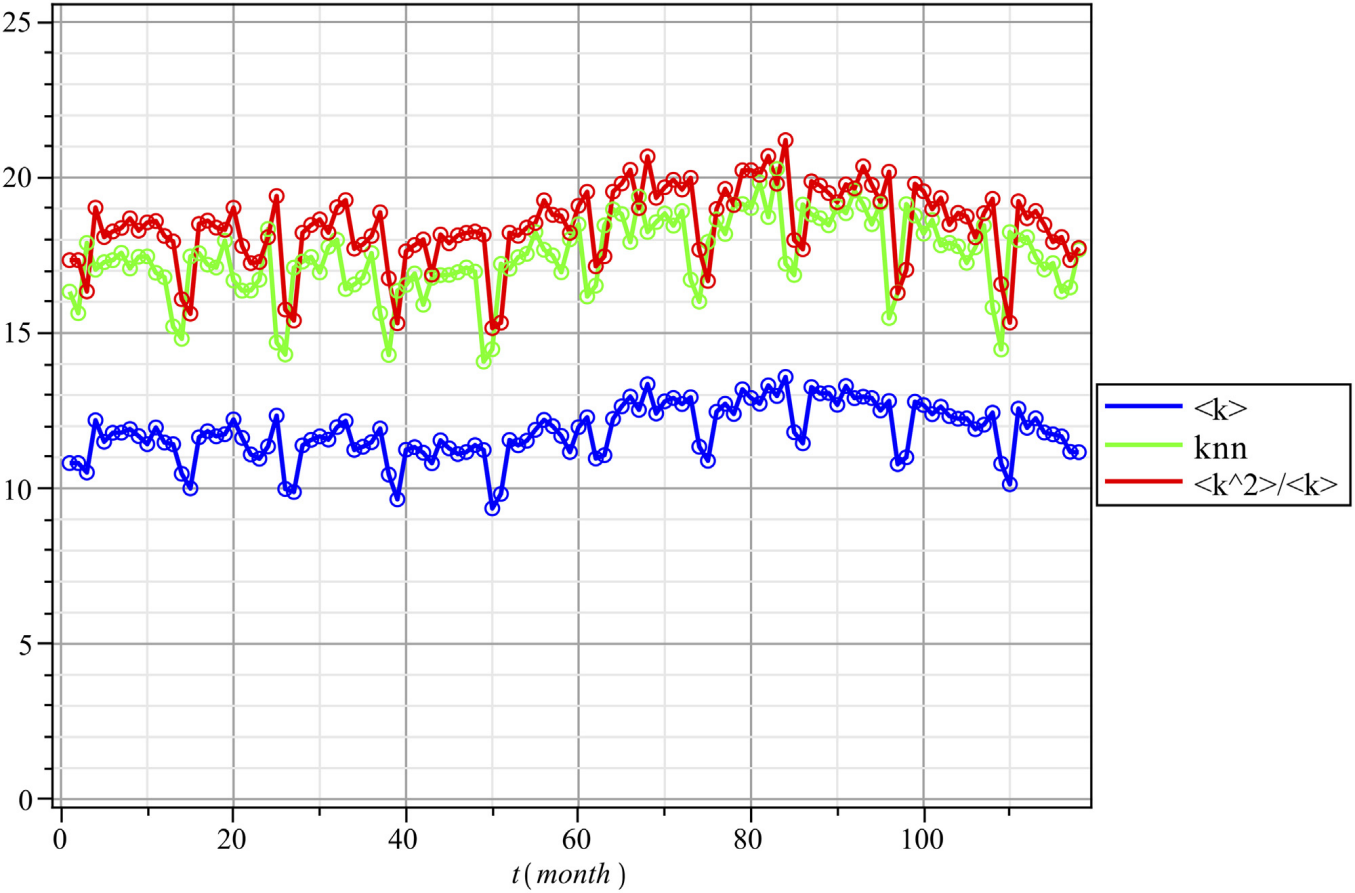
Average degree 〈*k*〉, average nearest neighbor degree *k*_*nn*_, and variance-to-mean ratio $\frac{\langle k^{2}\rangle }{\langle k\rangle }$ of the Network over Time and over Participating Vertices.

In Fig 5, we observe that $\frac{<k^{2}>}{<k>}\approx k_{nn}$. That is, the graph is *non-assortative* (there is proportionate or random mixing) for the degree.

To further corroborate proportionate mixing in the network, we used the *degree assortativity*, which can be measured as the Pearson correlation coefficient *r* [46, 47]:

$$\begin{array}{c} r=\frac{1}{\sigma_{q}^{2}}\sum_{jk}jk\left(e_{jk}-q_{j}q_{k}\right) \end{array}$$ (11)

where *q* is the degree distribution of a vertex at the end of a randomly chosen edge (i.e., degree distribution of nearest neighbors, as described previously); $\sigma_{q}^{2}$ is the sample variance of *q*; and *e*_*jk*_ is the joint probability distribution of the degrees of two connected vertices.

In a network with no assortative mixing, *q*_*j*_ and *q*_*k*_ are independent, and *e*_*jk*_ = *q*_*j*_ *q*_*k*_, so the coefficient is identically 0. We have 118 snapshots of an evolving network with degree assortativity ranging from 0.0068 to 0.0211 in our study. Therefore, we assert that we can consider this social network to be an evolving, non-assortative graph.

### 3.4 Estimating Values for SIS_*ϑ*_ Model Parameters

**Fig 6:**
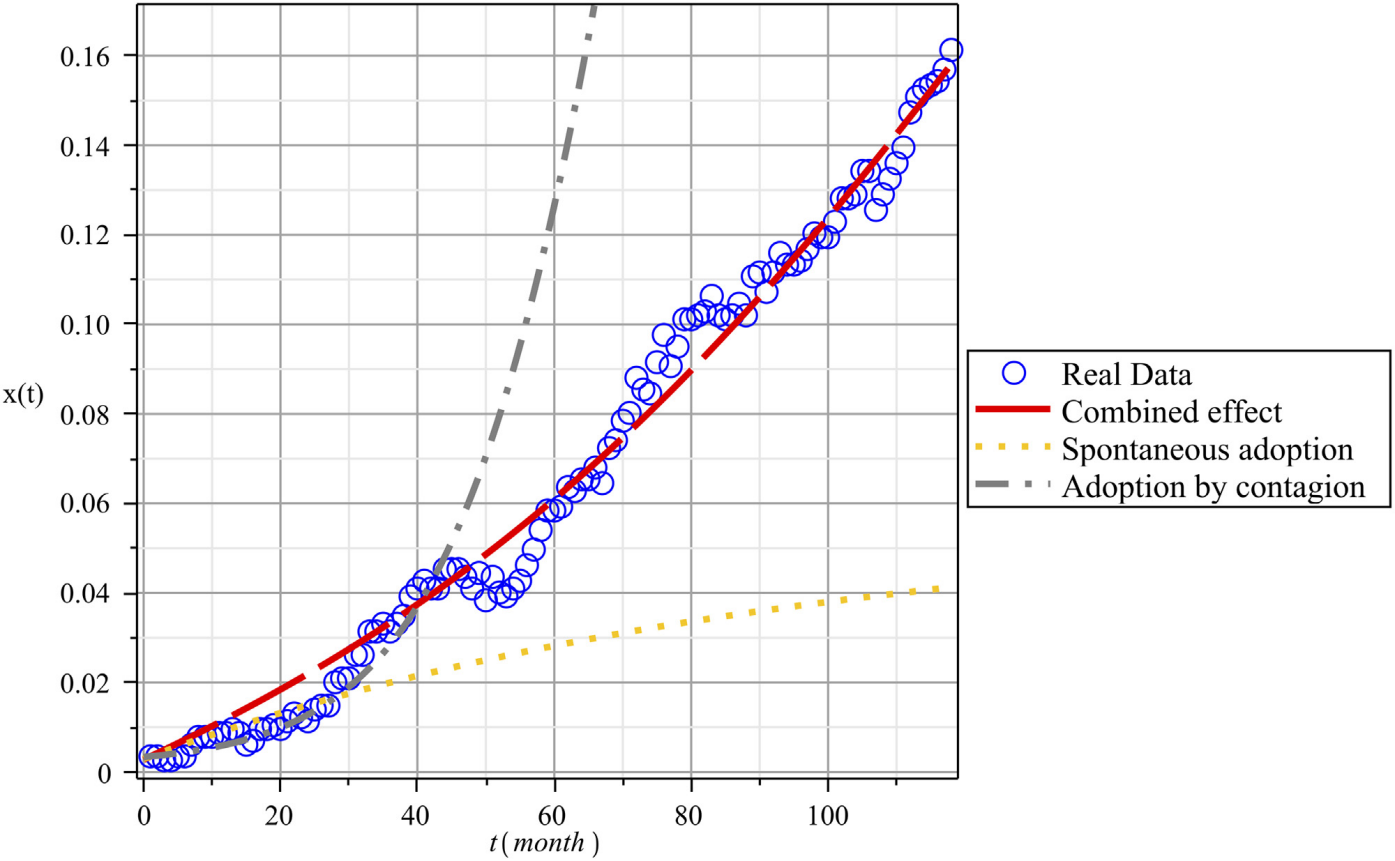
Fitting the Theoretical Solutions Provided by Model 2 and Adoption Data over Time. The gray curve represents the fit for the pure contagion case: *ϑ* = 0, *β* = 0.0045, *γ* = 0.01, with a residual mean square *SD* = 0.2. The orange curve is the fit for pure non-social influences: *ϑ* = 0.0006, *β* = 0, *γ* = 0.01, *SD* = 0.054622. The red curve is the fit for the combined effect *ϑ* = 0.0007, *β* = 0.0017, *γ* = 0.01, *SD* = 0.005.

First we consider the case of a static network. Fig 6 shows the fit for the three numerical solutions obtained from Model 2. Note that in this case the real data shown in Fig 6 is the accumulated data of the number of consumers who remained adopters until a given month. This is unlike the data shown in Fig 2, which records what happens in each specific month (month after month) regarding the number of adoptions and dis-adoptions of the Internet service.

The gray dash-dotted curve came from taking the corresponding spontaneous contagion parameter *ϑ* = 0. The values for the other parameters were found by fitting the solution given by the model and the actual evolution of adoptions in the community. The theoretical solution provided by the model in this case includes only the effect of social contagion through network links. As Fig 6 shows, we obtained a relatively good fit in this case for only the first 40 months, after which the model with these values for the parameters loses its predictive ability.

For the orange dotted curve, we set the parameter *β* = 0 and adjusted the parameter corresponding to spontaneous contagion *ϑ*. However, this yields an even worse fit than the previous option; after about 30 months, long-term predictions are impossible.

Finally, for the red dashed curve, we used two parameters, *β* and *ϑ*, to match the theoretical solution to real data. In this case, the model can reproduce the entire evolution of adoptions with notably better precision.

From this analysis, it seems clear that social contagion and non-social factors, such as advertising and influences from outside the community, play complementary roles in terms of their impact on the adoption process. Non-social influences most likely create new adopters spontaneously, whereas social contagion leads to the expansion of already existing adopters. The interplay of these complementary influences then gives shape to the dynamics of adoption.

In our empirical application we found that social contagion is approximately two and a half times stronger than non-social influence, $\frac{\beta }{\vartheta }\approx 2.5$. In other words, in the particular case of this community, members were more likely to adopt Internet service as a result of social influences than as a consequence of other, non-social factors.

### 3.5 Estimating the Values of the SIS_*ϑ*_ Model Parameters While Accounting for Social Dynamics

We apply Eq (5) to our data. For this time series, the temporal correlation coefficient is 0.5282. Using the metric defined in [34] produces a resultant temporal correlation coefficient of 0.3834.

**Fig 7:**
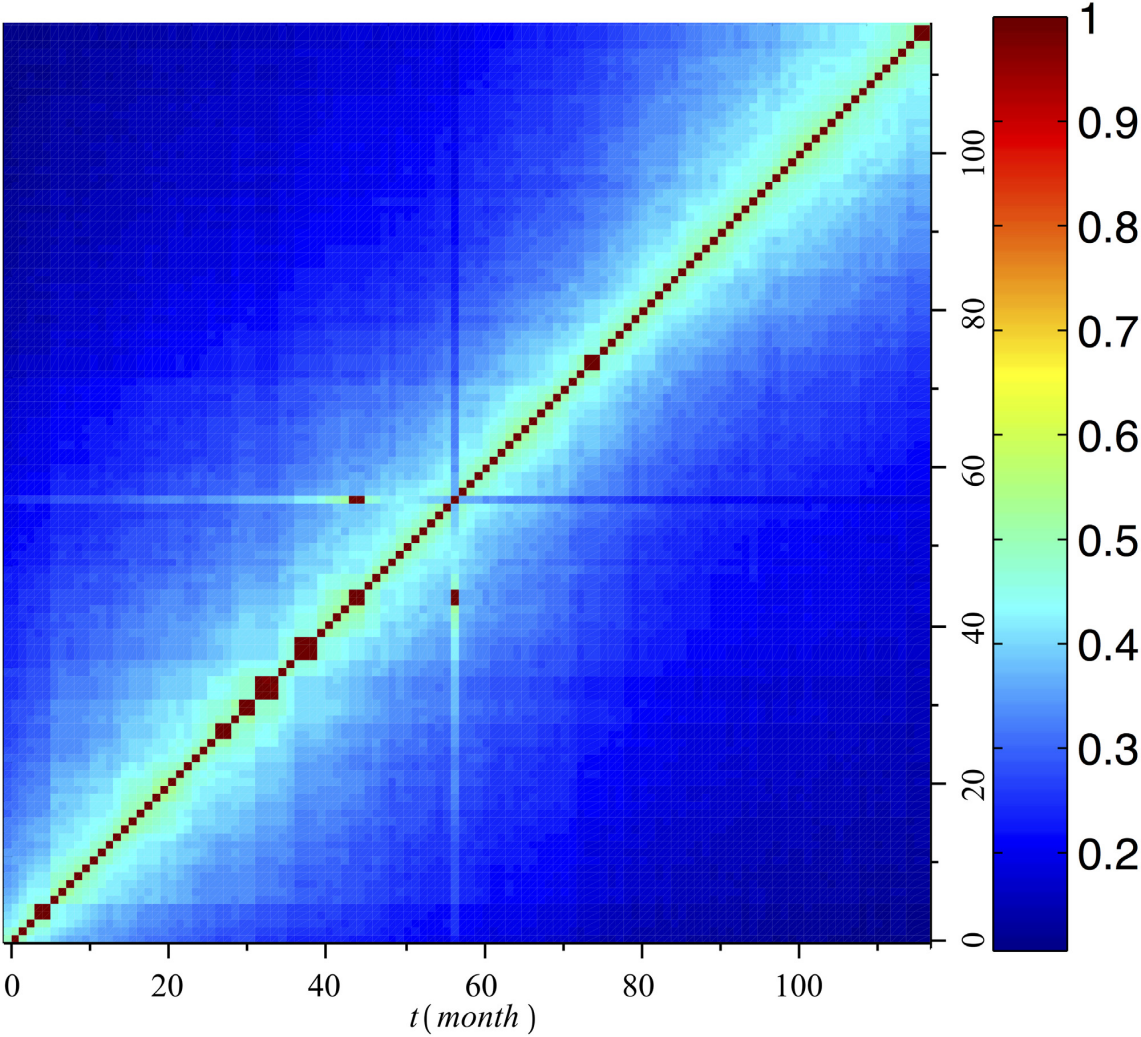
Topological overlap between every graph in the time series and every other graph in the time series.

The temporal correlation data reveal that as time passes, global changes in the graph progress evenly (see Fig 7). That is, the graphs do not change more in the beginning than at the end of the observed window (i.e., the gradient from red to navy is not greater at the bottom left than at the top right). Furthermore, as time progresses, the global features of the graphs overlap less and less. On average, the topological overlap between a graph and its temporal neighbor is 0.5282, which represents the temporal correlation coefficient. The average overlap is 0.2836 between any two graphs, and between the two most separated graphs (first and last), the topological overlap decreases to 0.1099, which constitutes the stable kernel of the series. However, these figures reflect only global tendencies. Each neighborhood of every vertex changes differently, at a different rate and with significant monthly fluctuations. It is also interesting to note that every vertex *i* has at least one point at which the temporal overlap between the neighborhood of *i*_*m*_ and *i*_*m*+1_ is equal to 0.

As mentioned in Section (1.3), we simulated the role of individually driven network dynamics in social contagion by using model (8), which includes network rewiring. This rewiring dynamics is independent of the contagion process. So, we first estimated the detachment and attachment rates from network data and then fit the parameters of contagion for the whole model. In this section we explain this procedure step by step.

It is worth noting that the network mean degree $\langle k\rangle =\frac{E}{N}$ is a conserved quantity: $\frac{d}{dt}\sum_{k}{\text{kP}}_{k}(t)=0$. Using this condition and master Eqs (6) and (7) we have:

$$\begin{array}{c} \sum_{k=1}^{\infty }P_{k}u_{k}=\sum_{k=0}^{\infty }P_{k}v_{k} \end{array}$$ (12)

We assume the rewiring depends only on the degree of the departure vertex. This simplification is consistent with indiscriminate rewiring and with the random mixing property of the network studied. In the particle analogy, when the particle hopping rate depends only on the occupation number of the departure site, the dynamics is called *Zero-Range Process* (ZRP) [42, 43]. For this case, introducing the notation *u*_*k*_ = *d*_*k*_ and using Eq (12) we have:

$$\begin{array}{c} u_{k}=d_{k},\;\;v_{k}=\sum_{l=1}^{\infty }P_{l}d_{l}=\langle d\rangle \end{array}$$ (13)

From Eq (6) and steady state condition $\frac{d}{dt}P_{k}(t)=0$ we get the relation:

$$\begin{array}{c} \frac{P_{k}}{P_{k+1}}=\frac{d_{k+1}}{\langle d\rangle } \end{array}$$ (14)

As the rewiring rate does not depend on the destination site, the end of the detached edge can be attached to any vertex in the network, so we use $v_{k}=\langle d\rangle =\frac{1}{N}$.

Using *v*_*k*_ so defined, and the steady state relation Eq (14) we can find the detachment rate:

$$\begin{array}{c} d_{k+1}=\frac{P_{k}}{NP_{k+1}} \end{array}$$ (15)

We can use relation Eq (15) directly to work with the detachment rate, but we can also estimate *d*_*k*_ by fitting the average degree distribution function from network data. Fitting *P*_*k*_ we obtain *P*_*k*_ ~ *k^a^e^−bk^*. The coefficients (95% confidence bounds) are *a* = 0.8199(0.7873, 0.8525), and *b* = 0.1548(0.1506, 0.1589). We substitute this fit of *P*_*k*_ in Eq (15) and get:

$$\begin{array}{c} d_{k}=\frac{{\text{e}}^{b}}{N}{\left(1-\frac{1}{k}\right)}^{a} \end{array}$$ (16)

Thus, we can rewrite the Eq (8) as follows:

$$\begin{array}{c} \frac{d}{dt}x_{k}=-\alpha \;x_{k}-\beta \;kx_{k}\Theta +\beta \;k\Theta +\vartheta -\;\nu \;x_{k}(d_{k}+\frac{1}{N})+\;\nu (\frac{x_{k+1}}{N}+d_{k}x_{k-1}) \end{array}$$ (17)

Where, we have used the Eq (15) and have introduced the parameter *ν*, which describes the overall time scale of the rewiring process, compared with that of the contagion process.

We use a heuristic approach for estimating model parameters and follow three basic steps: (1) We choose values for the parameters *β*, *ϑ*, *ν* from a mesh of equally spaced values (the value *γ* = *γ*_*s*_ is fixed, i.e. it is the same as in the case of a static network), (2) We solve the differential Eq (17) using these parameters, and (3) We calculate the minimum value using the least squares method:

$$\begin{array}{c} \min_{\beta ,\vartheta ,\nu }∥\;x(t)-\sum_{k=k_{\min}}^{k_{\max}}P_{k}x_{k}\left(\beta ,\vartheta ,\nu ,t\right)\;∥ \end{array}$$ (18)

Where, *x*(*t*) is the density of adopters obtained from data and *x*_*k*_(*β*, *ϑ*, *ν*, *t*) is the solution of the differential Eq (17) for the choosing parameters. The least squares method is applied using available data points for time *t*_*i*_ (month), *i* = 1, 2, …, *N*, with *T* = 118.

To choose the parameters we start the calculation with *ν* = 0, i.e. with a static network, and determine the values of *β*_*s*_ and *ϑ*_*s*_ for this static case. Then we build a three-dimensional grid with the intervals ∣*β* − *β*_*s*_∣ ≤ *h*_1_ *β*_*s*_, ∣*ϑ* − *ϑ*_*s*_∣ ≤ *h*_2_ *ϑ*_*s*_, and *ν* ∈ [0, 500]. *h*_1_ and *h*_2_ are some fractions of *β*_*s*_ and *ϑ*_*s*_ respectively. We used, several values for these parameters, but after some explorations we found reasonable results for *h*_1_ = *h*_2_ = 0.7 when *ν* ∈ [0, 500].

**Fig 8:**
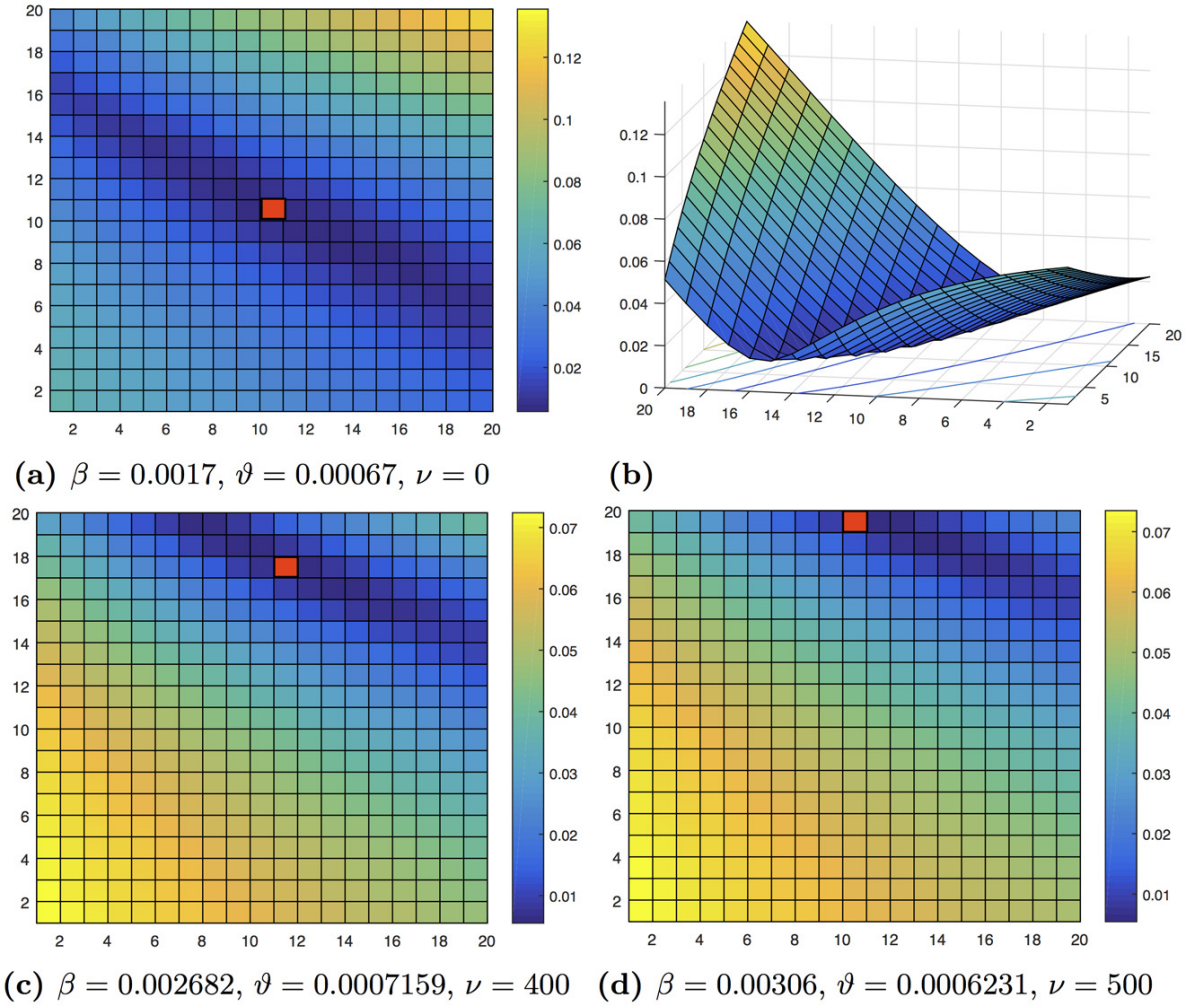
The figure shows an example of 20 × 20 mesh for fitting the model parameters. In (a), (c) and (d) the vertical axis indicates the interval partition for the values of parameter *β* and the horizontal axis indicates the partition for the parameter *ϑ*. A mesh is constructed for each value of the rewiring rate *ν*. The (*ϑ*, *β*) pair, which fulfills Eq (18), is represented by a red box in each figure. (a) depicts the case of a static network with *ν* = 0, and (b) is a three-dimensional representation of the fitting, where the vertical axis indicates the value found for the “distance” between the model solution and the real curve. Finally, (c) and (d) represent the cases of networks with rewiring.

**Fig 9:**
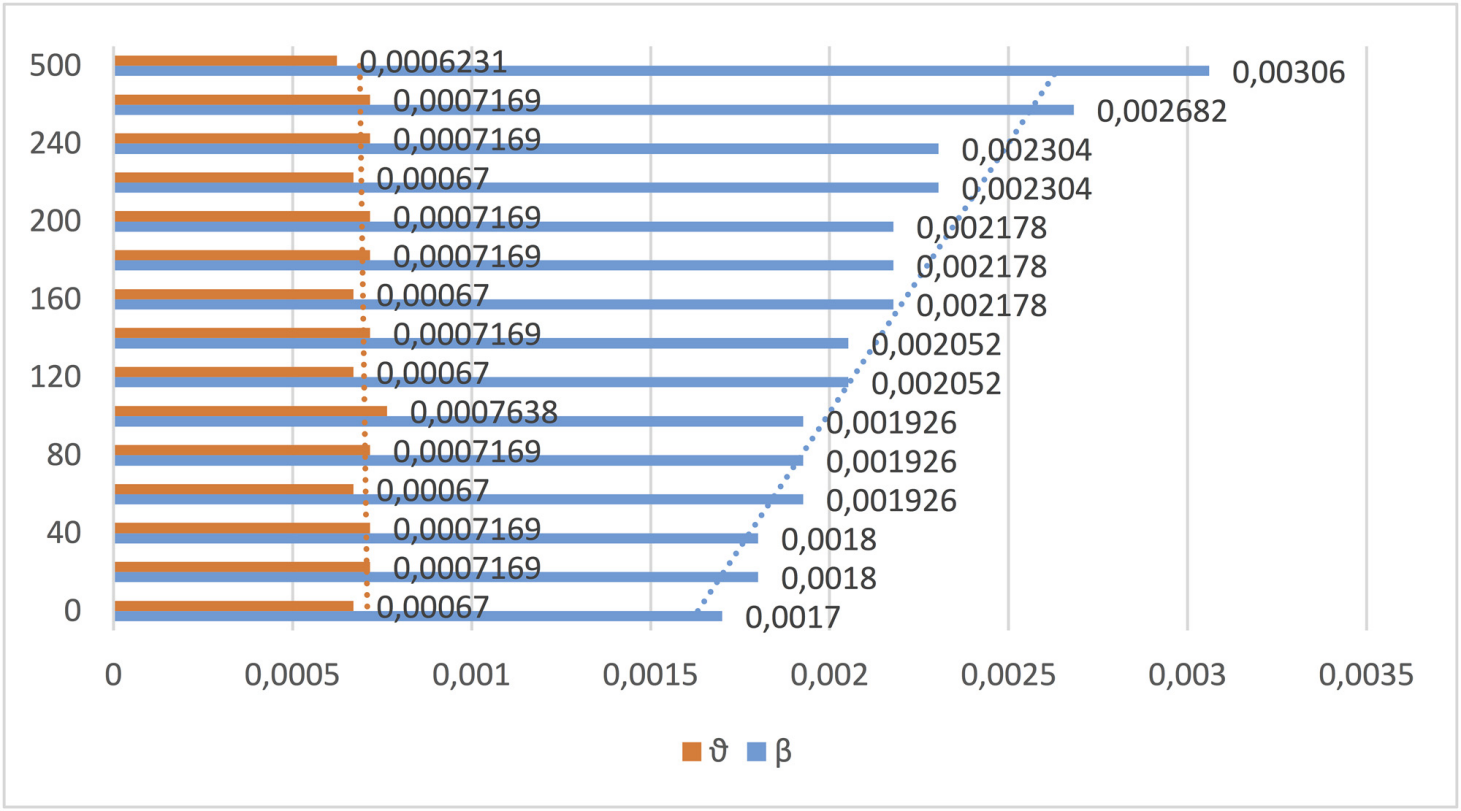
Values and trends for the parameters *ϑ* and *β* fitted for the model (17) for different rewiring rates *ν* = 0…500, corresponding to the curves represented in Fig 10.

Fig 8 depicts two important results. First, the network rewiring diminishes the contagion relative to the static case. Indeed, in order to keep the fit of the curve when rewiring occurs, the value of the *β* parameter should be adjusted upward. On the other hand *ϑ* oscillates between ranges close to its value from the static network. This behavior is shown more clearly in Fig 9. Second, we observe that the mesh used, which basically consists of analyzing the behavior of the network rewiring in a neighborhood of the static network, is useful for fitting the model’s parameters even for large values of the rewiring rate. The fitting procedures show great stability and clear trends in the parameter estimates.

**Fig 10:**
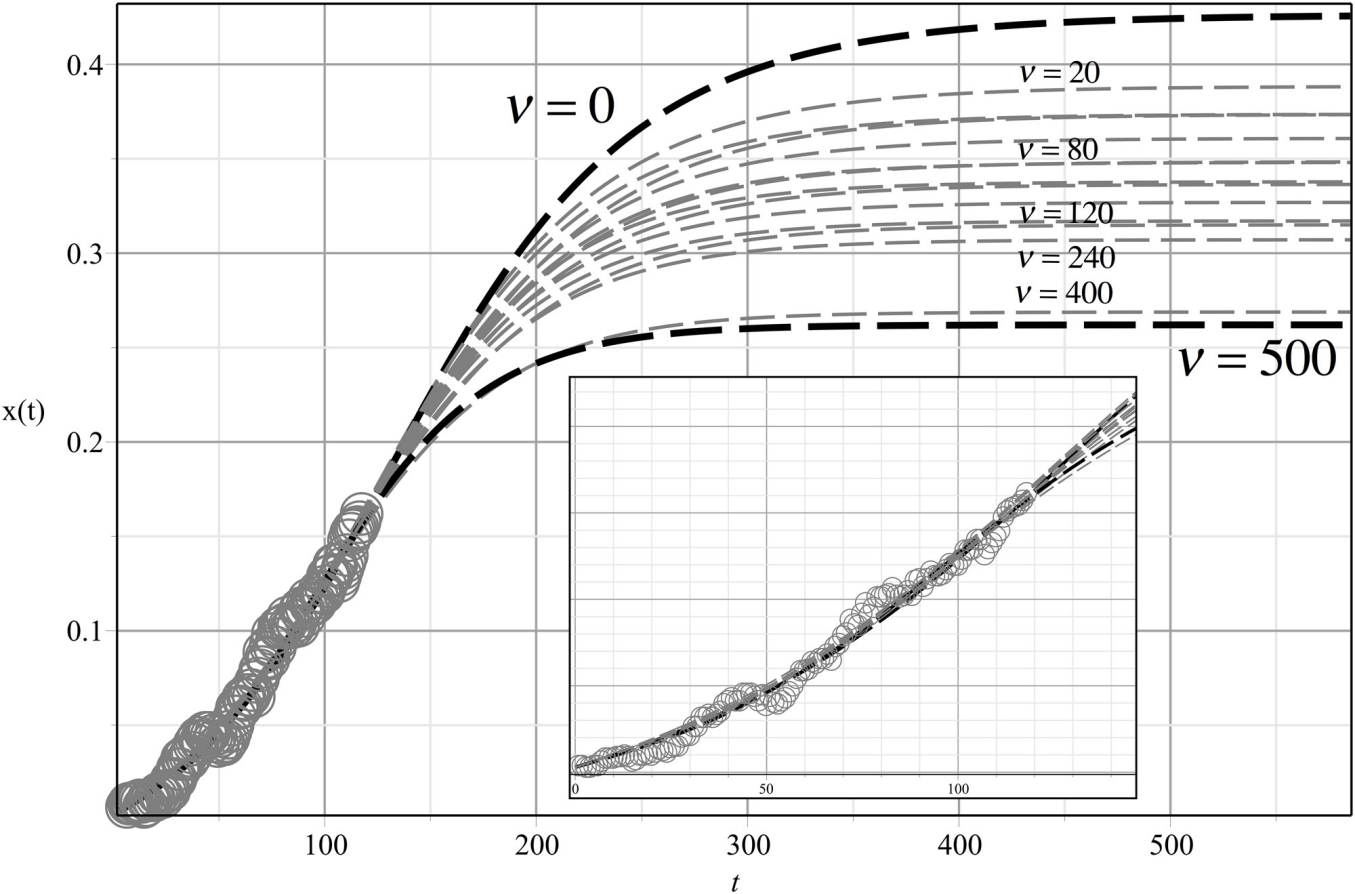
Long term prediction for adoption given by different rewiring rates *ν*. The fitting of available adoption data is replotted on a finer scale and is given in the inset. Note that increasing the rewiring reduces the adoption.

The contagion spreading is not necessarily enhanced by the dynamics of the network (see Fig 10), and the prevalence of contagion could be less than in the case of a static network. A plausible explanation for this behavior is as follows. In a static model, the only way to vary the number of connections each person has with others is by choosing a particular degree distribution for the network. However, the local shifting of the network affects the processes of contagion spread because the number of connections between individuals could eventually change.

In our empirical setting, the network has an (almost) fixed size, a fixed mean degree and even a fixed number of links, but it is not static since its structures vary over time. These changes in the number of connections between individuals can be modeled as rewiring processes. Because connections predispose individuals to contagion—causing contact, changes in the number of connections evidently will produce a variation in the spread of the contagion, because this contagion can only be transmitted along the links of the network and these links change over time.

**Fig 11:**
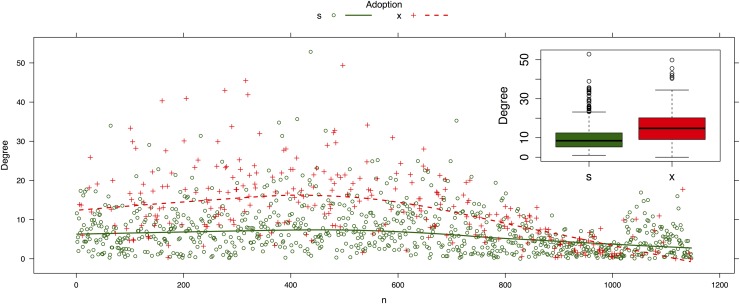
Mean degree of all network vertices sorted by adoption status. The red dots show infected vertices and the green dots represent susceptible vertices. The inset box chart clearly shows that the infected vertices have a higher degree.

Now we take the point of view of a susceptible vertex, and assume that this vertex has a link to an infected vertex. Usually, infected vertices have a higher degree than the susceptible ones because in heterogeneous networks a large fraction of hub vertices mediates the infection process (see for example [25, 48]). We corroborate this argument in Fig 11.

From Eq (16) we know that the detachment rate for links connected to hubs is higher than for low degree vertices. Thus, the link of this susceptible vertex with the infected one is quickly detached. Once a link is detached it is instantaneously attached to another vertex without preference by state of adoption or degree. Because the number of susceptible vertices is significantly higher than the number of infected vertices, which is more noticeable at the beginning of the diffusion of adoption, the probability that the link will be attached to another susceptible vertex is also high.

In summary, a combination of two factors explains why social contagion has a lower effect on adoption behavior for these dynamics networks: 1) the effective decrease of contagion rate because of the rapid detachment from infected vertices (usually, highly connected vertices) and 2) the non-preferential attachment to other vertices, mostly susceptible ones. Thus, the constant turnover of neighbor vertices could lead to an effective reduction of contagion and could even suppress the spread of contagion in the network, as was mentioned in [37, 48].

### 3.6 Results and Discussion

Traditional marketing instruments have become less effective [49], prompting marketers to seek new ways of influence customers. The increasing availability of network data in turn has triggered interest among managers in exploiting customers’ social networks. In this context, our research goal was to contribute to a better understanding of how and to what extent social contagion drives the adoption of a new product. Many studies have addressed this issue previously, using network theory [4, 5, 8, 9]. However, we take a novel approach and model social contagion by accounting for social dynamics over time, as well as for the multiple state changes from adoption to disuse that occur over the years. This consideration is critical, because actors in a social network exhibit different likelihoods of being influenced during the diffusion process, and this probability changes over time [9].

Rooted in theories of epidemiology, physics, and diffusion, we have developed a model to check how social contagion (WOM, imitation) and non-social factors (advertising, context) complement each other in terms of their impact on the adoption process. Furthermore, our model accounts for changes in social dynamics over time. We used a standard MF network SIS model and included the role of individual driven network dynamics in social contagion, according to network rewiring. We also checked the validity of our model by applying it to a unique data set that combines the dynamics of social relationships and adoption patterns among customers in a community over ten year period, with information gathered on a monthly basis. By studying a small community, we are sure that we have accounted for all relevant social relations among the individuals in our sample.

In line with previous research, the results of applying the model to our data reveal that both social contagion and contextual factors matter for the diffusion of innovations. The effect of either single factor alone (social interaction or contextual factors) cannot drive adoption by itself. However, social contagion prevails when we consider the weight that each factor has in terms of explaining adoption. In our empirical investigation, the social contagion coefficient is two and a half times greater than that of any of the other factors that affect adoption.

Despite the importance of social contagion, its effect weakens when we account for social changes over time. Our results indicate that using a single network over the diffusion period might overestimate the effect of social contagion on the adoption process. Specifically, we show empirically that in a static network, the adoption rate can reach 40% of the population. However, when we account for social dynamics this value drops to less than 30%.

This finding has meaningful managerial implications. In the age of the Internet and advanced communication technologies, both practitioners and scholars need to develop models that can help entrepreneurs and corporations leverage grass-roots marketing strategies and replace traditional marketing tools with WOM tactics for reaching consumers. Our results confirm that social contagion functions, but we also find that constant turnover among neighbors effectively reduces the rate of contagion and could even suppress its spread in the network. Therefore, practitioners need to be more cautious about assuming that social contagion has a strong effect, as some other researchers have warned as well [50]. With our empirical research, we demonstrate that the outcome depends mostly on the stability of networks over time. For that reason, and in line with the findings of Watts and Peretti in [51] and Van Den Bulte and Raghuram in [50], managers cannot simply stop investing in advertising during product launches. The complementary effect of free WOM depends substantially on the social network stability of the target community.

Our research also makes three further and related contributions. First, our model reveals a way to simplify network data, by using the degree distribution of the vertex instead of nodes. This approach reduces computation complexity for estimates of social contagion through social networks, which is relevant considering the size of the networks for most customers. Second, our method can be used to estimate the potential effect of social contagion on the adoption of new products. If firms can access their customers’ social network data (which is relatively easy, considering the availability of free public information stored in social network sites), they can simulate the expected diffusion of their new products through social contagion and test, for example, if first adopters are hubs or regular consumers, if firms should substantially increase their marketing effort at the beginning of the release campaign and target hubs directly, and so forth. Using this simple model, managers can assess whether grass-roots marketing campaigns are likely to work for the launching of their newest product, even before its release. However, it is worth noting that this implication will depend on network dynamics. If the rewiring process is too high, the strategy of targeting hubs would not work as expected because social contagion would be lower since the rewiring tends to homogenize the network.

Finally, the importance of relating the flexible, but abstract simulation-based approach with the more realistic, but limited empirical approaches is worth highlighting. For example Watts and Dodds [7] developed conditions under which opinion leaders might accelerate the diffusion of innovations while Goldenberg and colleagues [52] showed, using real data, that they might foster the diffusion of innovation depending on whether the targeted consumers are innovators or followers. Likewise, in our work we applied a simulation-based approach [11] for modelling the rewiring process in a real data set, extending some of the work of Schwarzkopf and colleagues.

### 3.7 Limitations and Further Research

We acknowledge some limitations in this study and thus some avenues for further research. First, our approach models social contagion as a single parameter, but social influence might be explained by several factors, such as WOM or peer pressure. It would be interesting to test the effect of different sources of social contagion in the diffusion process- with a more complex model and data that supports isolating the effect of each driver of social influence.

Second, and in line with our previous comment, we also model non-social factors as a single parameter, yet the weights of the different factors in the adoption process might differ. Additional research could address this problem by incorporating as many parameters as there are non-social issues that the research aims to test. Third, the simplicity of our model relies on the assumption that the empirical data (social networks) match the conditions required for MF theory and that the degree follows a given distribution. If these conditions are not met, researchers would need to use another approach.

In addition, future research might explore what-if scenarios in which simulation-based and empirical approaches are merged. An example of this line of work was done by Aral et al. [53] who studied conditions under which seeding and incentive strategies could work (simulation approach) in a real, large-scale social network of 27 million individuals interacting daily.

## Appendix

In the following we present the procedure to obtain Eq (8) for the SIS_*ϑ*_ model with rewiring.

Let $P_{k}^{\left(inf\right)}$ be the probability that a generic infected vertex has *k* edges. This probability is also the fraction of infected vertices of degree *k*. Due to the rewiring dynamics we have:

$$\begin{array}{c} \frac{d}{dt}P_{k}^{\left(inf\right)}(t)=u_{k+1}P_{k+1}^{\left(inf\right)}+v_{k-1}P_{k-1}^{\left(inf\right)}-(u_{k}+v_{k})P_{k}^{\left(inf\right)} \end{array}$$ (19)

If *X*_*k*_ is the number of infected vertices with degree *k* and *N*_*k*_ the number of vertices with degree *k*, we have the following relations:

$$\begin{array}{c} P_{k}^{\left(inf\right)}=\frac{X_{k}}{N}=\frac{N_{k}x_{k}}{N}=P_{k}x_{k} \end{array}$$ (20)

The next step is to substitute Eq (20) into Eq (19):

$$\begin{array}{c} \frac{d}{dt}\left(P_{k}(t)x_{k}(t)\right)=u_{k+1}P_{k+1}x_{k+1}+v_{k-1}P_{k-1}x_{k-1}-(u_{k}+v_{k})P_{k}x_{k} \end{array}$$ (21)

Now we consider the case in which the network is in a stationary steady state with respect to the rewiring dynamics, i. e. $\frac{d}{dt}P_{k}(t)=0$. The Eq (21) takes the following form:

$$\begin{array}{c} \frac{d}{dt}x_{k}(t)=u_{k+1}\frac{P_{k+1}x_{k+1}}{P_{k}}+v_{k-1}\frac{P_{k-1}x_{k-1}}{P_{k}}-(u_{k}+v_{k})x_{k} \end{array}$$ (22)

In an analogous manner we can obtain a similar equation for the density of susceptible vertices with degree *k*

$$\begin{array}{c} \frac{d}{dt}s_{k}(t)=u_{k+1}\frac{P_{k+1}s_{k+1}}{P_{k}}+v_{k-1}\frac{P_{k-1}s_{k-1}}{P_{k}}-(u_{k}+v_{k})s_{k} \end{array}$$ (23)

Similarly, if *S*_*k*_ is the number of susceptible vertices with degree *k* we have the following relations:

$$N_{k}=NP_{k}$$ (24)

$$N_{k}=S_{k}+X_{k}$$ (25)

$$1=\frac{S_{k}}{N_{k}}+\frac{X_{k}}{N_{k}}\Rightarrow s_{k}+x_{k}=1$$ (26)

Considering a stationary steady state with respect to the rewiring dynamics $\frac{d}{dt}P_{k}(t)=0$ we obtain:

$$\frac{dN_{k}}{dt}=N\frac{dP_{k}}{dt}$$ (27)

$$\frac{dP_{k}}{dt}=0\Rightarrow \frac{dN_{k}}{dt}=0\Rightarrow N_{k}\left(t\right)=N_{k}$$ (28)

$$1=s_{k}+x_{k}\;\forall \;k,\;t$$ (29)

The Eqs (22) and (23) take only the change in the densities of infected and susceptible vertices with degree *k* into account due to the rewiring dynamics. To consider the complete dynamics i.e. the *SIS*_*ϑ*_ model with rewiring, we should write:

$$\begin{array}{ccc} \frac{d}{dt}x_{k} & = & -\gamma x_{k}+\beta \;k\;s_{k}\Theta +\vartheta s_{k}-x_{k}(u_{k}+v_{k})+(\frac{x_{k+1}P_{k+1}u_{k+1}}{P_{k}}+\frac{x_{k-1}P_{k-1}v_{k-1}}{P_{k}})\\ \frac{d}{dt}s_{k} & = & \gamma x_{k}-\beta \;k\;s_{k}\Theta -\vartheta s_{k}-s_{k}(u_{k}+v_{k})+(\frac{s_{k+1}P_{k+1}u_{k+1}}{P_{k}}+\frac{s_{k-1}P_{k-1}v_{k-1}}{P_{k}}) \end{array}$$

It is easy to prove that these equations fulfil the normalization Eq (29). Indeed, summing both equations we have:

$$\begin{array}{c} \frac{d}{dt}(s_{k}+x_{k})=u_{k+1}P_{k+1}(s_{k+1}+x_{k+1})+v_{k-1}P_{k-1}(s_{k-1}+x_{k-1})-(u_{k}+v_{k})P_{k}(s_{k}+x_{k}) \end{array}$$ (30)

Using Eq (29) we write:

$$\begin{array}{c} 0=u_{k+1}P_{k+1}+v_{k-1}P_{k-1}-(u_{k}+v_{k})P_{k}=\frac{d}{dt}P_{k}(t)=0 \end{array}$$ (31)

From normalization Eq (29) we have *s*_*k*_ = 1 − *x*_*k*_, so, we can write the full equation (considering spontaneous infection, social contagion and rewiring dynamics) for the density of infected vertices in the following way:

$$\begin{array}{c} \frac{d}{dt}x_{k}=-\alpha \;x_{k}-\beta \;kx_{k}\Theta +\beta \;k\Theta +\vartheta -\;x_{k}(u_{k}+v_{k})+(\frac{x_{k+1}P_{k+1}u_{k+1}}{P_{k}}+\frac{x_{k-1}P_{k-1}v_{k-1}}{P_{k}}) \end{array}$$ (32)

where *α* = *ϑ* + *γ*.
